# Conditional neuromodulation of neurogenic detrusor overactivity using transrectal stimulation in patients with spinal cord injury: A proof of principle study

**DOI:** 10.1002/nau.23310

**Published:** 2017-06-30

**Authors:** Sarah Louise Knight, Nuwani Edirisinghe, Brian Leaker, Judith Susser, Michael Duncan Craggs

**Affiliations:** ^1^ London Spinal Cord Injury Centre Royal National Orthopaedic Hospital Stanmore UK; ^2^ Division of Surgery and Interventional Science University College London London UK; ^3^ Department of Medical Physics Bioengineering University College London London UK; ^4^ Nephro‐Urology Clinical Trials Ltd. Queen Anne Street Medical Centre London UK

**Keywords:** neurogenic detrusor over‐activity, neuromodulation, spinal cord injury

## Abstract

**Aims:**

A proof of principle study of a novel wearable device to control neurogenic detrusor over‐activity in eight male spinal cord injured subjects using conditional neuromodulation.

**Methods:**

Transrectal stimulation was delivered through the device in response to simultaneously recorded external anal sphincter (EAS) contraction as a marker for neurogenic detrusor overactivity (NDO). The effect of conditional neuromodulation on bladder capacity and maximum detrusor pressure was investigated in addition to reliability of dyssynergic sphincter contraction as a marker for NDO.

**Results:**

Conditional neuromodulation through the novel device showed a statistically significant increase in bladder capacity and reduction in maximum detrusor pressure in six male subjects with spinal cord injury (SCI). EAS activity was a reliable surrogate for detection of NDO.

**Conclusions:**

It has been shown for the first time that conditional neuromodulation can be delivered and triggered via a single biocompatible device placed in the anal canal. The pudendal nerves lying in Alcock's canal were stimulated through the wall of the anal canal, and the dyssynergic activity of the EAS was used to detect NDO and trigger neuromodulation giving significant increases in bladder capacity and reduction in detrusor pressure in six male subjects with SCI.

**Abbreviations**
AD: autonomic dysreflexia
CN: conditional neuromodulation
DP: detrusor pressure
DSD: detrusor sphincter dyssynergia
EAS: external anal sphincter
EAS EMG: external anal sphincter electromyogram
EMGthresh.min: value of EAS EMG at which CN would be triggered
EUS: external urethral sphincter
iSCI: incomplete spinal cord injury
MCC: maximum cystometric capacity
MDP: maximum detrusor pressure
NDO: neurogenic detrusor overactivity
PPV: positive predictive indicator
SCI: spinal cord injury
T_NDO_: time at which detrusor pressure exceeded 15 cmH_2_O

## 1 INTRODUCTION

Following a supra‐sacral spinal cord injury (SCI), there are profound alterations in bladder function which usually result in incontinence secondary to neurogenic detrusor overactivity (NDO) and associated detrusor sphincter dyssynergia (DSD).^1^ If left uncontrolled, these uninhibited reflexes may lead to impaired renal function as a result of reflux, incomplete emptying, and recurrent infection.

The ultimate goal in the urological management of patients with NDO and DSD is to increase bladder capacity and reduce bladder pressures, in order to protect the upper tracts and minimize incontinence. At present, this can be achieved using pharmacological methods, surgical procedures, or with the use of implanted electrical stimulation devices in combination with catheterization of the bladder.

A recent systematic review has shown that transcutaneous electrical stimulation is a safe and effective treatment for the neurogenic bladder, but the quality of evidence is low and many studies only have small numbers.^2^ Electrical stimulation (also known as neuromodulation) of the sacral, pudendal, and tibial nerves has been shown to effectively inhibit detrusor contractions caused by NDO.^2^, ^3^, ^4^, ^5^, ^6^, ^7^ This occurs through modulation of both residual supra‐sacral and segmental reflex pathways during storage and voiding phases of the micturition cycle.^8^, ^9^ The neuromodulation can be given continuously, or conditionally only when NDO occurs.

Continuous neuromodulation has been applied through surface electrodes on the dorsal penile or clitoral nerves, percutaneous electrodes on the tibial nerve^8^, ^9^, ^10^ and anal^11^ and intra‐vaginal plug electrodes.^12^ Implanted electrical stimulation devices (eg, Medtronic Interstim®) have also been used extensively to provide continuous neuromodulation of the sacral nerves in the treatment of neurogenic bladder dysfunction. A systematic review of sacral neuromodulation has shown that this treatment can be useful in selective cases.^13^

By contrast, conditional neuromodulation (CN) is applied only when NDO occurs using different mechanisms for detection of detrusor activity.^14^, ^15^, ^16^, ^17^, ^18^, ^19^, ^20^, ^21^ This has been achieved through recording of intra‐vesical pressure,^14^, ^15^ by using the dyssynergic activity of the urethral or anal sphincters^16^, ^17^, ^18^ or with more technical difficulty by recording from bladder sensory nerves.^19^ CN has been shown to be as effective as continuous neuromodulation in suppressing NDO and increasing bladder capacity but has the additional benefits of reducing both electrical power consumption and potential reflex habituation.^15^ Although CN appears to be a good alternative to continuous neuromodulation, none of the previous studies have developed beyond the laboratory into clinically applicable devices.

We describe a proof of principle study of a novel, patented,^20^ wearable, and biocompatible CN device to control NDO which overcomes some of the disadvantages of implanted continuous neuromodulation devices.

**Figure 1:**
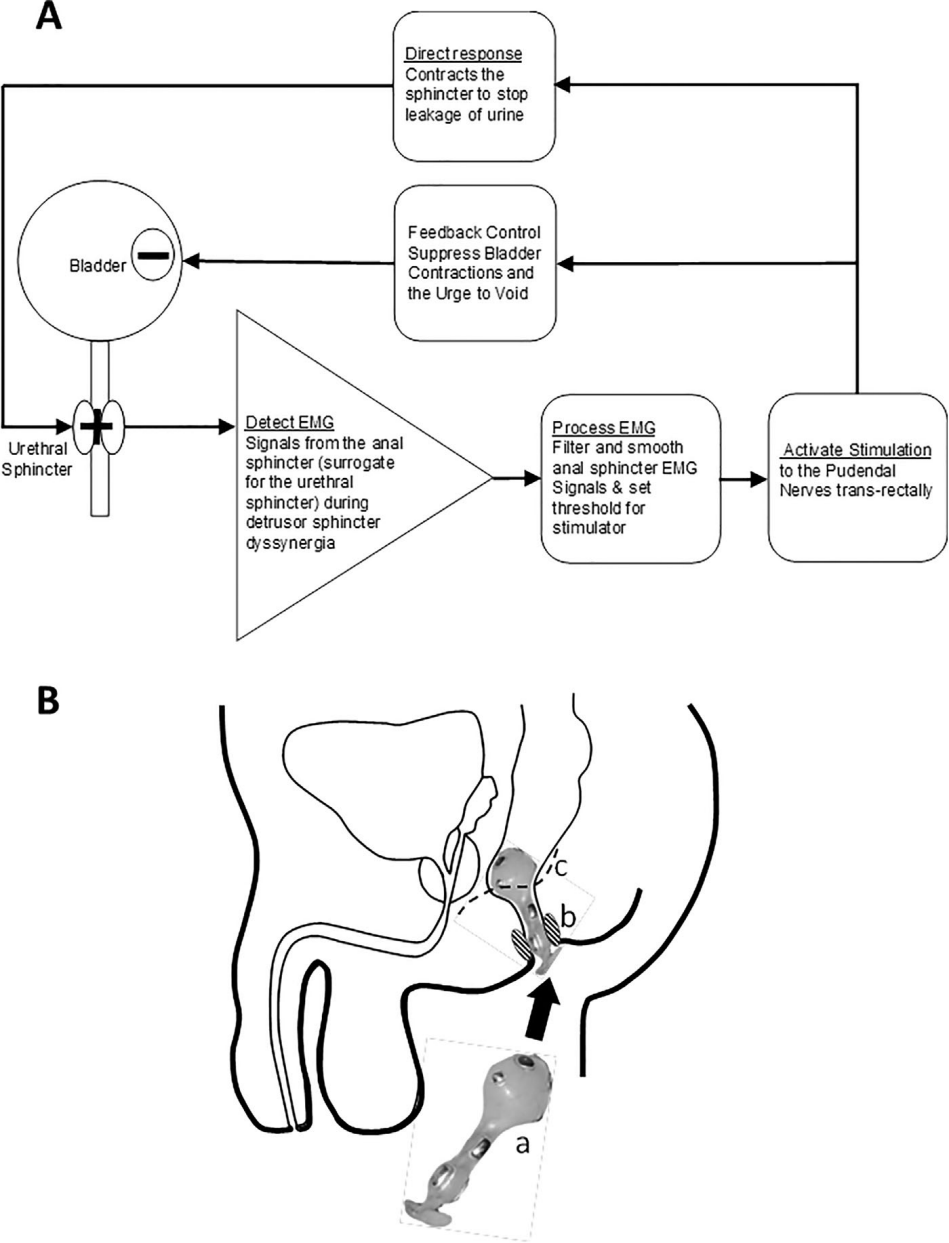
(A) Diagrammatic representation of conditional neuromodulation control in the CARM device. (B) The CARM device was manufactured from medical grade silicone rubber and shaped for conformity within the anal canal into which the device was inserted with the aid of electrode gel (a). The electrodes were manufactured using medical grade stainless steel. The EMG recording electrodes were located equi‐distant around the circumference to ensure best contact with the anal sphincter (b). The stimulating electrodes were designed as a bipolar pair bilaterally and directed toward the trajectory of the pudendal nerves (c) through Alcock's canal located near to the anorectal junction. A reference electrode was located at an electrically inactive position on the stem of the device

The conditional anorectal neuromodulation device (CARM) provides transrectal stimulation to the mixed pudendal nerves lying in Alcock's canal to suppress NDO. The stimulation is provided conditionally by simultaneously recording and utilizing the electromyographic activity of the co‐contraction of the external anal sphincter (EAS) as a trigger for the neuromodulation (Fig. 1A).

The aim of this study was to determine the efficacy of neuromodulation delivered through the CARM device and the reliability of using the activity of the dyssynergic anal sphincter as a marker for NDO in male subjects with SCI.

## 2 MATERIALS AND METHODS

The study protocol was approved by an independent Ethics Committee. The device was manufactured by the Department of Medical Physics, UCL. The study was conducted at the London Spinal Cord Injury Centre, Royal National Orthopaedic Hospital, Stanmore, UK.

### 2.1 Patient selection

Male subjects (age range 18‐75 years) with a complete or incomplete supra‐sacral SCI, of at least 24 months duration, were eligible to take part in the study. Subjects were excluded if they had any other neurological disorder, previous surgery to the bladder or sphincters, or previous intra‐detrusor botulinum toxin injections. They were also excluded if they showed positive leucocytes and nitrites on urinalysis on the day of investigation. All patients were requested to stop any anti‐muscarinic medication 7 days prior to investigation.

### 2.2 Standard cystometry

Standard filling cystometrograms were performed using 4.5 Ch catheters for measuring bladder pressure (Pves) and rectal pressure (Pabd); detrusor pressure (Pdet) was obtained by subtracting Pabd from Pves. Transducers were zeroed to atmosphere at the start of the investigation and placed at the level of the symphysis pubis, with patients in the supine position. The bladder was filled retrogradely with sterile saline through an additional 10 Fr filling line. For each cystometrogram maximum cystometric capacity (MCC), maximum detrusor pressure (MDP) and leakage were recorded.

### 2.3 Wearable conditional neuromodulation device

A prototype of the CARM device was developed for delivering CN (Fig. 1B). The device was controlled through a computer system with a CED power 1401 analog to digital converter (ADC), running Spike 2 software (Cambridge Electronic Design, Cambridge, UK). The stimulation was delivered from an electrically isolated constant current stimulator (DS7A, Digitimer, Welwyn Garden City, AL7 3BE, UK). The recording electrodes were connected to a Lectromed 5361 biopotential amplifier (Digitimer), the input EMG was AC coupled with a time constant of 0.03s, the signal was filtered using a digital band pass filter with cut off frequencies 60‐500 Hz, and then full wave rectified and smoothed with a time constant of 1 s. The customized data acquisition program enabled real‐time recording of cystometric pressures and sphincter EMG recordings. A threshold trigger for stimulation could be set on either the pressure or EMG channel.

The CARM device was inserted manually with lubricating medical gel into the rectum in the correct orientation. The correct position of the CARM device in situ is shown (Fig. 1B)

### 2.4 Baseline cystometrogram and EMG threshold determination

**Figure 2:**
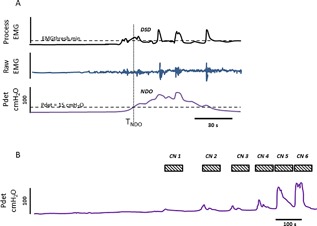
(A) An example of a typical baseline cystometrogram in this study showing the subtracted detrusor pressure (Pdet = Pves‐Pabd), the raw EAS EMG, and the processed EAS EMG activity recorded through anal sphincter electrodes on the CARM device. Maximum cystometric capacity (MCC) was defined as the volume at which there was urine leakage, 450 ml saline was infused, or the subject described discomfort. Maximum detrusor pressure (MDP) was also recorded. Raw and processed sphincter EMG recordings were used to determine threshold value for stimulation. The baseline cystometry was repeated three times in each subject in order to determine the repeatability of the parameters recorded. Subjects were excluded from the study if they did not demonstrate NDO or if the infusion volume exceeded 450 ml. The baseline CMG also shows a typical NDO contraction with associated DSD seen as an increase in EAS EMG activity. EMGthreshold was determined from the value of the processed EMG at the time that Pdet exceeded 15 cmH_2_O above baseline value (TNDO). The minimum of the three EMGthreshold values (EMGthreshold.minimum) was used as the trigger for subsequent conditional neuromodulation. (B) Typical detrusor pressure trace showing conditional neuromodulation through CARM device with multiple triggering of neuromodulation based on the activity in the anal sphincter. The stimulation parameters were optimized for each individual subject based on their sensory or motor threshold to stimulation through the device. For incomplete subjects, the sensory threshold was determined by the amplitude at which they could first feel a 5‐s burst of stimulation through the device. For complete subjects, the threshold was determined by the current at which a motor response (M‐wave) was elicited by a 30‐s burst of stimulation. The amplitude of the stimulating current was then set at twice this threshold. The stimulation frequency was set at 15 pps and pulse width 200 μs and was applied for periods of 60 s. The threshold for triggering conditional neuromodulation was set from the EMGthreshold.minimum value as described above. The number of stimulation bursts was recorded and for each suppressed NDO contraction, the peak detrusor pressure was measured. The time between each consecutive triggered stimulation was also recorded (Table 2). The MCC and MDP for conditional neuromodulation cystometrograms were also recorded

With the CARM device in situ, three baseline cystometrograms were performed at a filling rate of 60 mL/min in the supine position. Detrusor pressure and sphincter EMG were recorded simultaneously (Fig. 2A). The EMG threshold for each patient was determined by calculating the value of the processed EMG signal at the time that Pdet exceeded 15 cmH_2_O (T_NDO_) for each of the baseline fills. In order to reduce the incidence of false negatives, the minimum of the three values was used as the trigger for CN (EMGthreshold.minimum).

### 2.5 Conditional neuromodulation

CN was applied through the integral electrodes on the CARM, using biphasic balanced pulses. The amplitude of stimulation was determined for each subject based on twice sensory threshold or maximum that could be tolerated. A test fill at 60 mL/min was carried out in each subject to determine whether a single burst of CN could suppress NDO and be tolerated. Subjects were excluded from the study if NDO was not suppressed at maximum tolerated stimulation amplitude.

The bladder was then re‐filled at a physiological rate of 15 mL/min (Fig. 2B) with CN applied through the CARM. A 60‐s train of stimulation was triggered each time EMGthreshold.minimum was reached. The end point of the experiment was determined as when CN could no longer suppress NDO, a volume of 500 mL was reached or there was leakage.

### 2.6 Statistical analysis

The Wilcoxon sign rank test was used to compare MCC and MDP at baseline and during CN.

## 3 RESULTS

### 3.1 Patient demographics

Twelve male subjects with SCI were recruited into the study. Following baseline cystometrogram, four were excluded as they did not demonstrate NDO or their bladder capacity exceeded 450 mL. Four subjects were classified with ASIA Impairment Score (AIS) complete (A) and four were incomplete (B‐D). The level of injury, year of injury, AIS, and bladder management were recorded (Table 1).

**Table 1:** Table showing demographic details of the eight patients in the study detailing level of injury, date of injury, ASIA Impairment Score, and current bladder management

					Baseline (mean and SD of three fills)		Conditional neuromodulation			
Patient no.	Level of injury	ASIA impairment score	Date of injury	Bladder management	MCC (mL)	MDP (cmH_2_O)	MCC (mL)	No. of contractions suppressed	Mean peak DP (cmH_2_O)	MDP (cmH_2_O)
P1	T11‐T12	B	2006	ISC+AM	152 ± 3	57 ± 3	195	2	42	56
P2	T10‐T11	A	2005	ISC+AM	97 ± 9	86 ± 10	488	3	54	72
P3	C3‐C7	D	2002	ISC+AM	155 ± 17	100 ± 9	530	4	68	73
P4	T4	A	2007	IC+AM	80 ± 25	106 ± 19	510	7	51	69
P5	T12	D	1998	ISC+pads	231 ± 50	138 ± 35	289	3	34	41
P9^a^	C6‐C7	D	2000	ISC+AM	241 ± 25	57 ± 7				
P11	T3‐T4	A	2001	ISC	386 ± 28	79 ± 4	405	4	45	48
P12^a^	T2	A	1987	ISC+AM	429 ± 83	79 ± 15				

ISC, intermittent self‐catheterization; AM, anti‐muscarinic medication; IC, indwelling catheter. Mean baseline cystometric data from eight patients entered into the study. Cystometric data (MCC and MDP) for six patients who underwent conditional neuromodulation through CARM device.

^a^ Patients excluded from study during conditional neuromodulation. P9 did not show successful suppression of NDO which may have been due to insufficient stimulation current, as he was unable to tolerate higher than 25 mA. P12 experienced autonomic dysreflexic symptoms so was excluded from further study.

### 3.2 Baseline cystometry and sphincter EMG

The results of the baseline cystometry (MCC and MDP) are shown in Table 1. A corresponding increase in EAS EMG was observed in each subject who demonstrated NDO, consistent with the presence of DSD. The EMG threshold.minimum values are shown in Table 2.

**Table 2:** Table showing mean EMGthreshold values for three baseline cystometrograms and EMGthreshold.minimum value used for subsequent conditional neuromodulation fill

					Negative		Positive			
Patient no.	Mean EMG at Pdet = 15 cmH_2_O (μV)	EMG threshold min (μV)	Stimulation threshold (mA)	Stimulation current (mA)	True	False	True	False	Unwanted stimulation %	PPV
P1	8 ± 4	5.2	30	57	–	–	2	–	–	1.0
P2	3 ± 1	2.3	30	60	–	–	9	–	–	1.0
P3	11 ± 4	8.3	30	60	–	–	13	5	14	0.7
P4	5 ± 3	3.5	30	60	–	–	16	2	6	0.9
P5	35 ± 2	32.4	3	40	–	–	6	4	22	0.6
P9	31 ± 5	25.4	20	25						
P11	6 ± 2	4.6	50	90	–	–	6	3	15	0.7
P12	10 ± 2	8.1	40	80						

The stimulation threshold (motor or sensory) and the actual stimulation current is also shown. The number of true and false positive and negative stimulations for the conditional neuromodulation fill are also shown with the number of % of unwanted stimulation and the positive predictive indicator (PPV).

### 3.3 Conditional neuromodulation

**Figure 3:**
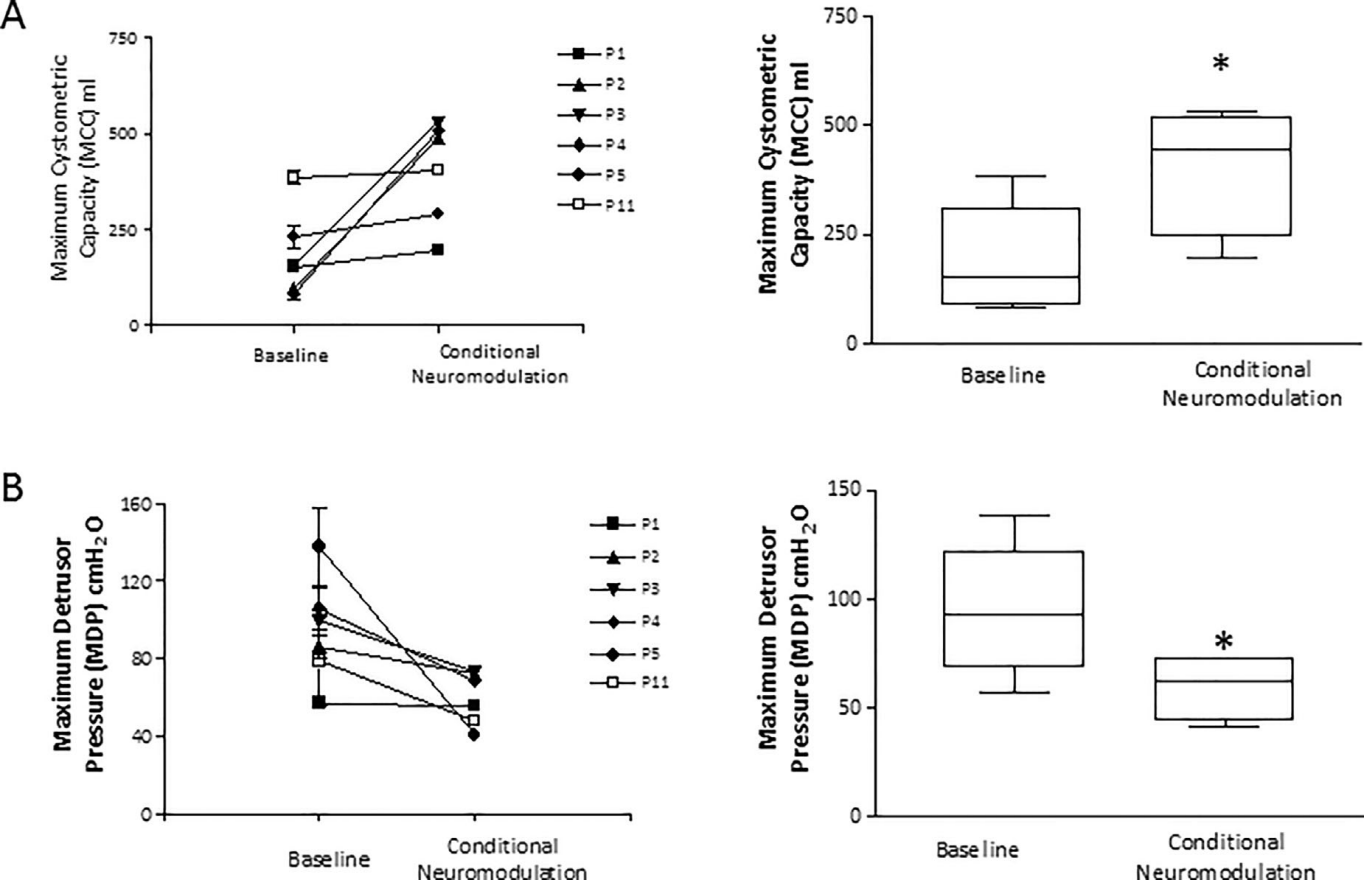
(A) Box and whisker graph representing the median (and interquartile range) of maximum cystometric capacity (MCC) during baseline and conditional neuromodulation fills for six subjects. Error bars denote range. The graph to the left shows the individual values for MCC at baseline and during conditional neuromodulation. The * indicates a *P* value <0.03 following a Wilcoxon sign rank test. (B) Box and whisker graph representing the median (and interquartile range) of maximum detrusor pressure (MDP) during baseline and conditional neuromodulation fills for six subjects. Error bars denote range. The * indicates a *P* value <0.03 following a Wilcoxon sign rank test. The graph to the left shows the individual values for MDP at baseline and during conditional neuromodulation

**Figure 4:**
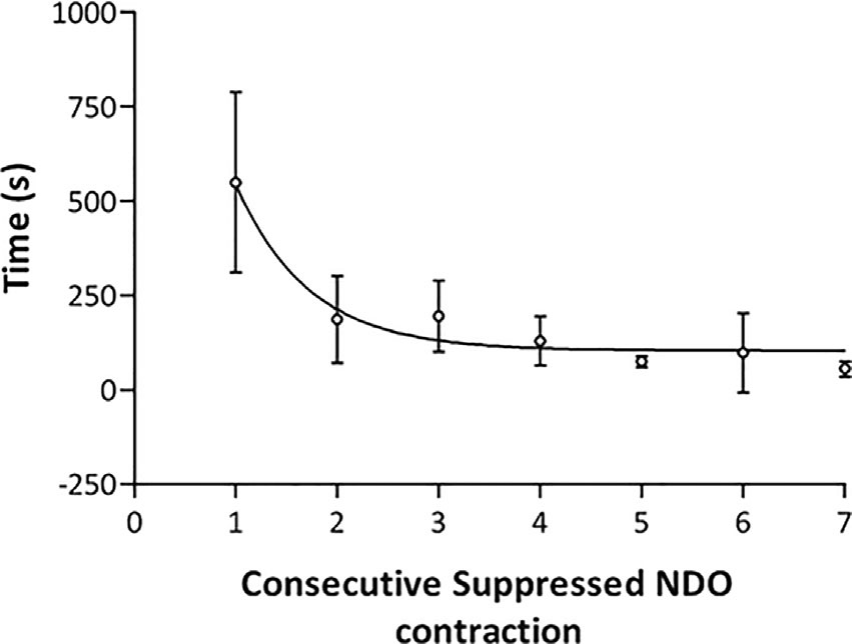
The mean time between each consecutive detrusor contraction for six subjects during conditional neuromodulation. The regression line shows a one‐phase exponential decay with a time constant of 0.49 and *r* ^2^ of 0.98

A typical trace of CN through the CARM device is shown in Fig. 2B. Two subjects were excluded from the study after the trial CN; one subject with AIS D could not tolerate the required level of stimulation to suppress NDO and the second (AIS A) experienced slight AD symptoms (headache and flushing) during filling. The stimulation currents and number of suppressed contractions are shown in Table 2, with a mean current of 62 ± 16 mA. There was a statistically significant increase in MCC from a median (interquartile range) value of 153 (88‐308) milliliter at baseline to 446 (242‐530) milliliter during CN (Fig. 3A). There was a statistically significant decrease in MDP from 93 (68‐122) cmH_2_O at baseline to 62 (44‐72) cmH_2_O during CN (Fig. 3B). The mean number of suppressed detrusor contractions was 4 ± 2. The time interval between detrusor contractions became shorter as the bladder capacity increased, until the neuromodulation could no longer suppress them. The time intervals were fitted with a one‐phase exponential decay curve which had a half‐life of 0.49 (Fig. 4).

### 3.4 Positive predictive value for EAS EMG activity

During the CN fills, using the EMGthreshold.minimum to trigger stimulation, false positive and false negative detections were calculated for each patient (Table 2). There were no false negative detections during the CN fills. The average number of true positives throughout the CN fills was 9 (±5) and the average number of false positives was 2 (±2). The mean positive predictive value (PPV) for the series of fills was 0.8 (±0.2) for the six patients.

## 4 DISCUSSION

This investigation was a proof of principle study of a novel wearable device to control NDO in male subjects with SCI using CN. The CARM device, worn in the ano‐rectum delivers neuromodulation to the pudendal nerves lying in Alcock's canal. The neuromodulation was delivered conditionally, in response to simultaneously measured EMG activity in the EAS. The EMG EAS activity corresponded well with NDO. The results of this pilot study in six SCI subjects with NDO showed that CN through the CARM device statistically significantly increased MCC and significantly reduced MDP. This study has shown for the first time that CN can be delivered and triggered through a single device worn in the anal canal.

The device and procedure were well tolerated in the majority of the subjects; one subject with an incomplete lesion could not tolerate the stimulation through the device required to suppress NDO.

Neuromodulation has been used in many previous studies to suppress NDO in patients with supra‐sacral SCI.^2^, ^3^, ^4^, ^5^, ^6^, ^7^, ^8^, ^9^, ^10^, ^11^, ^12^, ^13^, ^14^, ^15^, ^16^, ^17^ using a variety of different electrode sites. Typical stimulation parameters which give effective detrusor inhibition have been well documented.^14^ Optimal frequency ranges are given as 5–50 Hz. Very low frequencies can be associated with unpleasant sensations, so 10–15 pps are normally used. Balanced biphasic pulses with pulse width 200–500 μs are commonly used to minimize electrochemical reactions at the electrode‐tissue interface. The amplitude of stimulation must be sufficient to elicit activity in the relevant nerves, but not so high as to cause painful sensations. The stimulation amplitude is therefore normally determined for each individual patient with a minimum level of twice the motor threshold for the pudendo‐anal reflex in complete patients.^14^ In the current study, a stimulation frequency of 15pps and pulse width of 200 μs was used. The range of stimulation currents was between 40 and 90 mA with a mean of 61.16 mA, the incomplete subjects had lower stimulation currents than the complete subjects reflecting the lower sensory thresholds. Only one subject (iSCI) could not tolerate stimulation current high enough to suppress NDO. Therefore the parameters required when stimulating the pudendal nerves lying in Alcock's canal are similar to those used in surface stimulation of the dorsal penile/clitoral nerve, and were well tolerated even in the incomplete patients. A direct comparison with the currents used through implanted devices such as the Medtronic Interstim is not possible as the settings are not easily determined and success in SCI patients is limited.

CN has previously been described using detrusor pressure as a trigger for stimulation.^14^, ^15^, ^16^ Although CN has been shown to be as effective as continuous neuromodulation in increasing bladder volume and reducing detrusor pressure, it has only to date been carried out under laboratory conditions, as long‐term remote pressure measurement within the bladder is not possible. Fjorback et al^21^ described a portable device which was based around ambulatory urodynamics and DPN stimulation which demonstrated that CN was effective at increasing bladder capacity by suppressing NDO in patients with SCI in a more physiological setting than repeated laboratory fills. However, this technique could still only be used as a screening tool rather than a treatment in itself, until a non‐invasive technique for measuring bladder pressure is developed.

Therefore, an alternative to detrusor pressure as a trigger for CN was required. Blaivas et al^1^ have described the relationship between the external urethral sphincter (EUS) and detrusor activity following SCI. They described three types of DSD, in which the EUS co‐contracts with the detrusor muscle during NDO contractions. The dyssynergic sphincter contractions are supplied by the pudendal nerve which innervates the skeletal muscles in the pelvic area; it has been shown in cats and humans that the EAS also contracts during dyssynergic contractions of the EUS.^22^, ^23^ Three potential signals are therefore available as a trigger for detecting onset on NDO; pudendal nerve activity (ENG), EUS EMG and EAS EMG. Wenzel et al^22^ have described the use of pudendal nerve ENG in the spinalized cat model to trigger CN to suppress NDO contractions successfully but the technique has not yet been translated to a device for use in humans. The EUS EMG is only recordable using needle electrodes which are invasive and can be uncomfortable as well as not being practical for long‐term monitoring. By contrast, the EAS EMG can be readily recorded from an electrode placed in the anal canal as described in the current study.

The use of the dyssynergic activity of the EAS during NDO contractions has been speculated by previous authors. Horvath et al^16^ used EAS EMG to trigger dorsal penile nerve stimulation and showed similar increases in bladder capacity with both conditional and continuous stimulation, but with much reduced stimulation times when using CN. They reported that there were no false negative detections of NDO. Wenzel et al^17^ describe a retrospective study of urodynamic traces of 47 patients and prospective study of 79 patients of which 81 had SCI, which showed that signal processed EAS EMG could be a reliable indicator of NDO contraction. They also used a closed loop system using the EAS as a trigger for CN using dorsal penile nerve stimulation in the cat model which successfully detected and suppressed NDO contractions. Both authors report that there may be some false positive stimulation which would create an open loop rather than closed loop system but that false positives are better than false negatives. Indeed, in our study, we have shown that there were no false negative stimulations and a mean positive predictive value (PPV) for the series of fills was 0.81 (±0.17) for the six subjects. The incidence of false positives may increase when the device is used on a day‐to‐day basis due to artifacts in the EAS EMG or reflex activity of the sphincter. Enhanced EMG signal processing and better optimization of individual thresholds may decrease the incidence of false positives.

Using the wearable device, we have described significant increases in bladder capacity during CN triggered by EAS EMG activity. Median (interquartile range) baseline bladder capacity in the six patients was 153 (88‐308) milliliter which increased to 446 (242‐530) milliliter following CN representing a more than a twofold increase in bladder capacity. These results are consistent with the findings of Kirkham et al^14^ and Dalmose et al^15^ who also used slow filling rates but used bladder pressure to trigger CN. The MDP during the CN was also significantly reduced when compared to control fills and therefore would provide protection of the upper tracts. Sub‐group analysis between subjects with complete and incomplete SCI was not possible due to the small sample size, but of the six subjects who were analyzed, three were AIS A and three were AIS B‐D.

During CN, the time between each successive NDO contraction was recorded until the neuromodulation could no longer effectively suppress the contractions. The time between each contraction was observed to decrease as the bladder volume increased following a one‐phase exponential decay with a half‐life of 0.49. This decrease in time between consecutive contractions has also been noted by other researchers using CN^17^ and it has also been shown in the spinal cat that the frequency of unstable contractions increases with bladder volume.^24^ The decay in time between successive contractions could be used to notify the patient when their maximum bladder capacity is approached, allowing them to empty their bladder before any leakage occurred.

### 4.1 Study limitations and outlook

As this was a proof of principle study, it is recognized that one of its limitations was the small number of subjects tested. In addition, of the eight subjects undergoing neuromodulation, two could not tolerate the stimulation through the device and were withdrawn before the final part of the study. One subject with an incomplete injury could not tolerate the required strength of stimulation and one developed symptoms of autonomic dysreflexia following stimulation. This may limit the device use in some patients, but careful pre‐assessment would identify these patients as with other neuromodulation devices. We are currently redesigning the device to improve electrode design^25^ and seeking a manufacturer to develop it further and carry out longer term safety, efficacy, and tolerability trials in a larger population group. We believe this device could offer an alternative to implantable neuromodulation devices in some patients, and may eventually benefit patients with other neurological and non‐neurological detrusor overactivity

## 5 CONCLUSIONS

It has been shown for the first time that CN can be delivered and triggered via a single device placed in the anal canal. The pudendal nerves lying in Alcock's canal were stimulated through the wall of the anal canal, and the dyssynergic activity of the EAS was used to detect NDO and trigger neuromodulation. Significant increases in bladder capacity and decreases in maximum detrusor pressure were demonstrated in a group of six male spinal cord injured subjects.
